# iCartiGD: the Integrated Cartilage Gene Database

**DOI:** 10.1186/1471-2156-8-4

**Published:** 2007-02-23

**Authors:** Ming-Yiu Yeung, David K Smith, Matthew SY Chan, Cheuk M Li, Brian C Wong, Kenneth MC Cheung, Keith DK Luk, Kathryn SE Cheah, Pak Sham, Danny Chan, You-Qiang Song

**Affiliations:** 1 Department of Biochemistry, Li Ka Shing Faculty of Medicine, The University of Hong Kong, 21 Sassoon Rd, Pokfulam, Hong Kong, China; 2 Department of Orthopaedics and Traumatology, Li Ka Shing Faculty of Medicine, The University of Hong Kong, 21 Sassoon Rd, Pokfulam, Hong Kong, China; 3 Department of Psychiatry, Li Ka Shing Faculty of Medicine, The University of Hong Kong, 21 Sassoon Rd, Pokfulam, Hong Kong, China; 4 Genome Research Centre, Li Ka Shing Faculty of Medicine, The University of Hong Kong, 21 Sassoon Rd, Pokfulam, Hong Kong, China

## Abstract

**Background:**

Diseases of cartilage, such as arthritis and degenerative disc disease, affect the majority of the general population, particularly with ageing. Discovery and understanding of the genes and pathways involved in cartilage biology will greatly assist research on the development, degeneration and disorders of cartilage.

**Description:**

We have established the Integrated Cartilage Gene Database (iCartiGD) of genes that are known, based on results from high throughput experiments, to be expressed in cartilage. Information about these genes is extracted automatically from public databases and presented as a single page report via a web-browser. A variety of flexible search options are provided and the chromosomal distribution of cartilage associated genes can be presented.

**Conclusion:**

iCartiGD provides a comprehensive source of information on genes known to be expressed in cartilage. It will remain current due to its automatic update capability and provide researchers with an easily accessible resource for studies involving cartilage. Genetic studies of the development and disorders of cartilage will benefit from this database.

## Background

Diseases of cartilage, such as arthritis and degenerative disc disease, affect the majority of the general population, particularly with ageing. In recognition of the impact of musculo-skeletal disorders on society, 2000–2010 has been declared the bone and joint decade by the World Health Organization [1]. One aim of this decade is to foster research relevant to musculo-skeletal systems. Discovery and understanding of the genes and pathways involved in cartilage biology will greatly assist research on the development, degeneration and disorders of cartilage. To this end we have established the Integrated Cartilage Gene Database (iCartiGD) of genes that are known, based on results from high throughput experiments, to be expressed in cartilage. Information about these genes is extracted automatically from public databases and presented as a single page report via a web-browser. Several flexible search options are provided and the chromosomal distribution of cartilage associated genes can be presented. iCartiGD provides researchers with an easily accessible resource for studies involving cartilage.

When compared with databases of relatively well studied organ systems, such as the human prostate gene database [2] or the ovarian kaleidoscope database [3], databases of cartilage associated genes are less developed or not publicly available. A skeletal gene database [4] and its accompanying skeletal transcript database have been created. These databases contain a limited number of genes and approximately 80,000 ESTs mainly from human and mouse trabecular bone and bone marrow stromal cell libraries. Recently an Osteo-Promoter Database has been created [5] which contains information on the promoter regions of the approximately 600 genes in SGD. Both these databases provide links to other sources but do not give comprehensive reports on the genes.

However, large amounts of information about cartilage associated genes are available publicly. Numerous genes involved in skeletal development have been discovered through *in vitro *and *in vivo *studies [6,7]. Expressed sequence tag (EST) libraries, prepared from both normal and diseased human cartilage have been created [8-10] as have cDNA libraries [11]. Another similar set of libraries from Serial Analysis of Gene Expression (SAGE) also provides cartilage specific expression libraries. Microarray based studies of cartilage tissue have also been conducted [12,13]. These libraries provide lists of genes that are expressed in various cartilage tissue subtypes and indicate their level of expression and their degree of differential expression between diseased and normal cartilage tissue. iCartiGD combines these data with other gene specific information such as its nomenclature, chromosome position, sequence, protein domains or families, homologs, SNPs, expression levels in various tissues, gene ontology, associated disorders and literature references.

iCartiGD has been designed to facilitate access to the wealth of publicly available information on cartilage associated genes by providing a one-stop source for this information. Rather than search a variety of databases, scientists and clinicians studying cartilage can utilise the automatically updated iCartiGD, with its flexible search functions, to access the data and resources they require.

## Construction and content

Many databases of publicly available information have been mined or cross-linked and integrated to create iCartiGD. These databases include several of the National Center for Biotechnology Information (NCBI) databases such as Genbank, Entrez Gene, UniGene, MapViewer, dbEST, dbSNP, HomoloGene, SAGEmap, PubMed, Gene Expression Omnibus (GEO), RefSeq and OMIM. Other resources and databases used are Ensembl, UniProt, the UCSC genome browser, the Protein Data Bank (PDB), InterPro, Sage Genie, HapMap, Affymetrix, SymAtlas of the Genomics Institute of the Novartis Research Foundation, the Gene Ontology Annotation project (GOA), and the Kyoto Encyclopedia of Genes and Genomes (KEGG). See Table 1 for a list of their web addresses.

**Table 1:** URLs of databases and resources integrated via iCartiGD

Database/Resource	URL
NCBI	
Entrez Gene	
UniGene	
MapViewer	
HomoloGene	
dbEST	
SAGEmap	
PubMed	
OMIM	
GEO	
RefSeq	
dbSNP	
Ensembl	
UniProt	
PDB	
Sage Genie	
InterPro	
HapMap	
Affymetrix	
SymAtlas	
UCSC genome browser	
GOA	
KEGG	

Only genes that are expressed in cartilage are included in the database. We determine genes that are expressed in cartilage based on reference to four lines of evidence. Currently, eight EST libraries from dbEST at the NCBI give approximately 5,000 genes that are expressed in normal and osteoarthritic cartilage, and chondrosarcomas. SAGE libraries derived from chondrosarcomas, and microarray studies and a cDNA library of normal cartilage complete the cartilage tissue data set. This gives a total of approximately 14,000 genes (based on EntrezGene identifiers) that are expressed in at least one cartilage tissue type. An evidence display, based on these sources, reports the justification for the inclusion of a gene in the database.

EntrezGene records were primarily used to build the database. Where appropriate, UniGene or Affymetrix probe-ids were mapped to EntrezGene identifiers. Based on these, the relevant information from the databases mentioned above was downloaded and integrated into iCartiGD. The gene name, obtained via the EntrezGene identifier, forms the primary key for the tables in iCartiGD, with the Human Genome Nomenclature Commission (HGNC) gene name being used where available.

Data parsing and extraction from the source databases are performed by PERL scripts, some of which utilise published parser modules [14]. The extracted data are entered into tables in a MySQL database. PHP scripts are used to generate HTML web pages dynamically for the graphical user interface. Web pages can also be generated in XML format and transformed by XSL so that iCartiGD is web-services ready. The bioinformatics server of the BIOSUPPORT project of the University of Hong Kong hosts iCartiGD. This server also provides regularly updated local mirrors of many of the source databases from which iCartiGD is automatically updated on a weekly basis.

Update of the database is performed by automatically regenerating the database each week using a shell script to link the data extraction and database update scripts. The EST and other libraries are down loaded for each update run and the weekly updated mirrors of the source databases provided by the BIOSUPPORT project are accessed. This allows iCartiGD to take account of frequent modifications to databases such as UniGene and to provide the correct mappings from UniGene to the latest version of EntrezGene. Rebuilding the database from the current versions of the source databases ensures all modifications to the data are obtained.

## Utility and discussion

**Figure 1:**
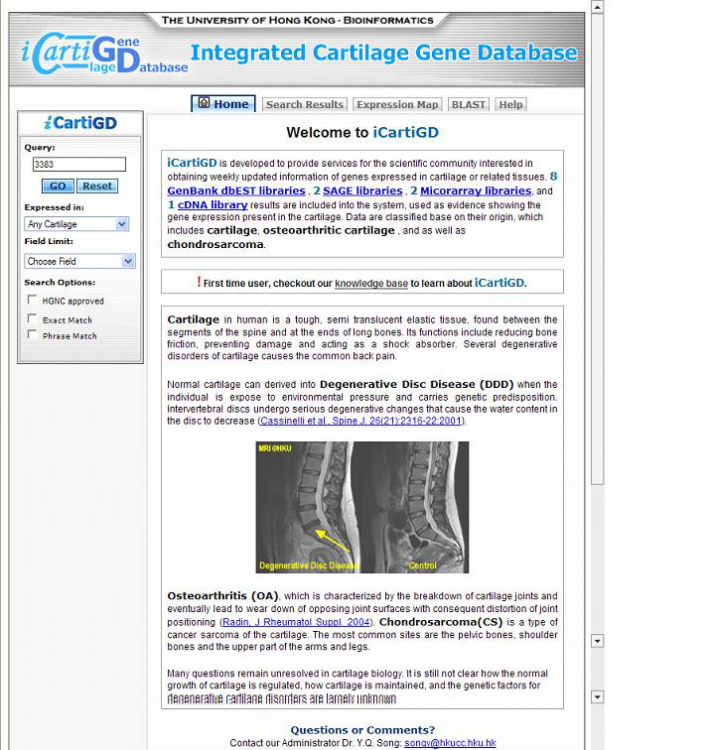
The home page of iCartiGD.

An ever expanding amount of information that would be of interest to biologists and clinicians who study cartilage is being gathered in a wide range of databases. If these data are to be utilised effectively, coordination of the resources should prove an invaluable help to the research community. iCartiGD has been developed to provide a single point of entry for cartilage biologists into this wealth of knowledge. A unified web-based, graphical user interface (GUI) was developed to allow users to search and access the data in iCartiGD (Figure 1). Currently over 14,000 genes that are known to be expressed in cartilage tissue types have been stored in iCartiGD, thereby allowing a comprehensive report of the information recorded about them to be readily retrieved by cartilage researchers. Not only does this report collate the available information on the gene but it also allows the user to link into the source databases to search further if required.

For cartilage researchers needing access to information on their genes of interest, a variety of query options have been provided through either a quick query box or a query form. The query form allows the combination of search results or further searches within the current results, and the history of the last 30 searches is retained. Queries of the database can be based on all genes in the database, or on the genes expressed in a particular tissue subtype. Advanced search options, such as field limits, Boolean operators, wild cards and phrase matching are available. It is possible for a user to construct queries in URL format so that scripts can be used to access the database instead of the web-based GUI if high throughput is needed. Users can also browse the genes stored in the database by chromosome number or alphabetically by the gene symbol or the gene name. Another form of query is provided by an interface to the BLAST search programs [15]. Users may search for matches to the sequences in iCartiGD using either the nucleic or amino acid sequences of interest to them.

To assist with genetic studies, the genomic location of the genes and transcripts in iCartiGD can be visualised on a transcriptome map. At any chromosomal position a user can retrieve the genes or transcripts in the database at that location. This will allow the identification of genes that are expressed in cartilage in the region of, for example, a candidate gene for cartilage disease, or the cartilage expressed genes in a region in significant linkage with disease markers. Comparisons of the differences in gene expression patterns among the cartilage tissue types can be made by examining the gene or transcript density [16] along each chromosome. Clustering within the genome of cartilage expressed genes can also be examined by tissue subtype with this facility.

As iCartiGD is automatically updated on a weekly basis from major public data sources it will not suffer the lack of currency that befalls many databases. In the event of new EST libraries or large scale transcriptome studies on cartilage being made available, they can be added to the gene expression sources parsed by the update scripts which extract the list of gene identifiers to be supplied to update iCartiGD. Current data for the newly obtained and existing genes will then be automatically retrieved and included in iCartiGD.

A knowledgebase is provided in the website to allow new visitors to become familiar with site. Questions received from different individuals can be posted in the knowledge base when appropriate to also allow fellow users to respond to queries or initiate discussions.

Searches of expression databases and the literature will be used to identify new studies of gene expression in cartilage. Further developments in iCartiGD will include a differential display tool to assist with comparative studies of different conditions and tissue subtypes. Improved methods to search the database using finer subdivisions of the data than the tissue sub-type currently available will be added. Other methods to examine the genomic distribution of cartilage expressed genes [17] will be included, as will genes associated with cartilage disorders, as they are identified from our and others' ongoing studies.

## Conclusion

iCartiGD provides a comprehensive source of information about genes known to be expressed in cartilage. It will remain current due to its automatic update capability and will facilitate the research of basic scientists and clinicians studying cartilage related genes. Genetic studies of cartilage development and disorders will also benefit from this database.

## Availability and requirements

iCartiGD is available to the public at 

## Authors' contributions

YQS initiated and supervised the project; MYY, MSYC, CML and DKS carried out the database construction and programming; DKS wrote the manuscript from an initial draft by MYY; DKS, BCW, KMCC, KSEC, PS, DC and YQS participated in its design and coordination and helped to revise the manuscript. All authors read and approved the final manuscript.

## References

[B1] Bjdoline (World Health Organization). http://www.boneandjointdecade.org.

[B2] Li LC, Zhao H, Shiina H, Kane CJ, Dahiya R (2003). PGDB: a curated and integrated database of genes related to the prostate. Nucl Acids Res.

[B3] Ben-Shlomo I, Vitt UA, Hsueh AJW (2002). Perspective: The Ovarian Kaleidoscope Database-II. Functional Genomic Analysis of an Organ-Specific Database. Endocrinology.

[B4] Jia L, Ho NC, Park SS, Powell J, Francomano CA (2001). Comprehensive resource: Skeletal gene database. Am J Med Genet.

[B5] Grienberg I, Benayahu D (2005). Osteo-Promoter Database (OPD) - promoter analysis in skeletal cells. BMC Genomics.

[B6] Karsenty G, Wagner EF (2002). Reaching a genetic and molecular understanding of skeletal development. Dev Cell.

[B7] Kronenberg HM (2003). Developmental regulation of the growth plate. Nature.

[B8] Jung YK, Jeong JH, Ryoo HM, Kim HN, Kim YJ, Park EK, Si HJ, Kim SY, Takigawa M, Lee BH, Park RW, Kim IS, Choi JY (2004). Gene expression profile of human chondrocyte HCS-2/8 cell line by EST sequencing analysis. Gene.

[B9] Kumar S, Connor JR, Dodds RA, Halsey W, Van Horn M, Mao J, Sathe G, Mui P, Agarwal P, Badger AM, Lee JC, Gowen M, Lark MW (2001). Identification and initial characterization of 5000 expressed sequenced tags (ESTs) each from adult human normal and osteoarthritic cartilage cDNA libraries. Osteoarthritis Cartilage.

[B10] Zhang H, Marshall KW, Tang H, Hwang DM, Lee M, Liew CC (2003). Profiling genes expressed in human fetal cartilage using 13,155 expressed sequence tags. Osteoarthritis Cartilage.

[B11] Pogue R, Sebald E, King L, Kronstadt E, Krakow D, Cohn DH (2004). A transcriptional profile of human fetal cartilage. Matrix Biol.

[B12] Yager TD, Dempsey AA, Tang H, Stamatiou D, Chao S, Marshall KW, Liew CC (2004). First comprehensive mapping of cartilage transcripts to human genome. Genomics.

[B13] Olney RC, Wang J, Sylvester JE, Mougey EB (2004). Growth factor regulartion of human growth plate chondrocyte proliferation in vitro. Biochem Biophys Res Commun.

[B14] Liu M, Grigoriev A (2005). Fast parsers for Entrez Gene. Bioinformatics.

[B15] Altschul SF, Madden TL, Schaffer AA, Zhang J, Zhang Z, Miller W, Lipman DJ (1997). Gapped BLAST and PSI-BLAST: a new generation of protein database search programs. Nucl Acids Res.

[B16] Qiu P, Benbow L, Liu S, Greene JR, Wang L (2002). Analysis of a human brain transcriptome map. BMC Genomics.

[B17] Li Q, Lee BT, Zhang L (2005). Genome-scale analysis of positional clustering of mouse testis-specific genes. BMC Genomics.

